# Correction: In vivo treatment with a non-aromatizable androgen rapidly alters the ovarian transcriptome of previtellogenic secondary growth coho salmon (*Onchorhynchus kisutch*)

**DOI:** 10.1371/journal.pone.0323020

**Published:** 2025-04-24

**Authors:** Christopher Monson, Giles Goetz, Kristy Forsgren, Penny Swanson, Graham Young

The word “Oncorhynchus” is misspelled in the article title. The correct title is: In vivo treatment with a non-aromatizable androgen rapidly alters the ovarian transcriptome of previtellogenic secondary growth coho salmon (*Oncorhynchus kisutch*). The correct citation is: Monson C, Goetz G, Forsgren K, Swanson P, Young G (2024) In vivo treatment with a non-aromatizable androgen rapidly alters the ovarian transcriptome of previtellogenic secondary growth coho salmon (*Oncorhynchus kisutch*). PLoS ONE 19(10): e0311628. https://doi.org/10.1371/journal.pone.0311628
